# Radiological manifestation of optic nerve infarction in a patient with invasive mucormycosis secondary to diabetic ketoacidosis in Pakistan

**DOI:** 10.1002/ccr3.6869

**Published:** 2023-01-19

**Authors:** Shehroze Tabassum, Aroma Naeem, Afshan Shakir, Faiza Afzal, Laya Ohadi

**Affiliations:** ^1^ Department of Internal Medicine King Edward Medical University Lahore Pakistan; ^2^ Department of Radiology King Edward Medical University Lahore Pakistan; ^3^ School of Medicine Shahid Beheshti University of Medical Sciences Tehran Iran

**Keywords:** diabetic ketoacidosis, mucormycosis, optic nerve infarction

## Abstract

A 35 years old male patient presented in the hospital with complaints of left‐sided facial swelling, blindness in the left eye, and left eye proptosis. He had a concomitant history of diabetic ketoacidosis. Magnetic resonance imaging was advised, which revealed infected tissue of the left cheek, optic nerve infarction, intracranial extension, and leptomeningeal involvement by the disease process.

## Abstract 2

Immunocompromised patients usually suffer from fungal infections. If not managed in time, patient can end up having optic nerve infarction and invasive intracranial complications.

## 1 CLINICAL IMAGE AND DESCRIPTION

**FIGURE 1:**
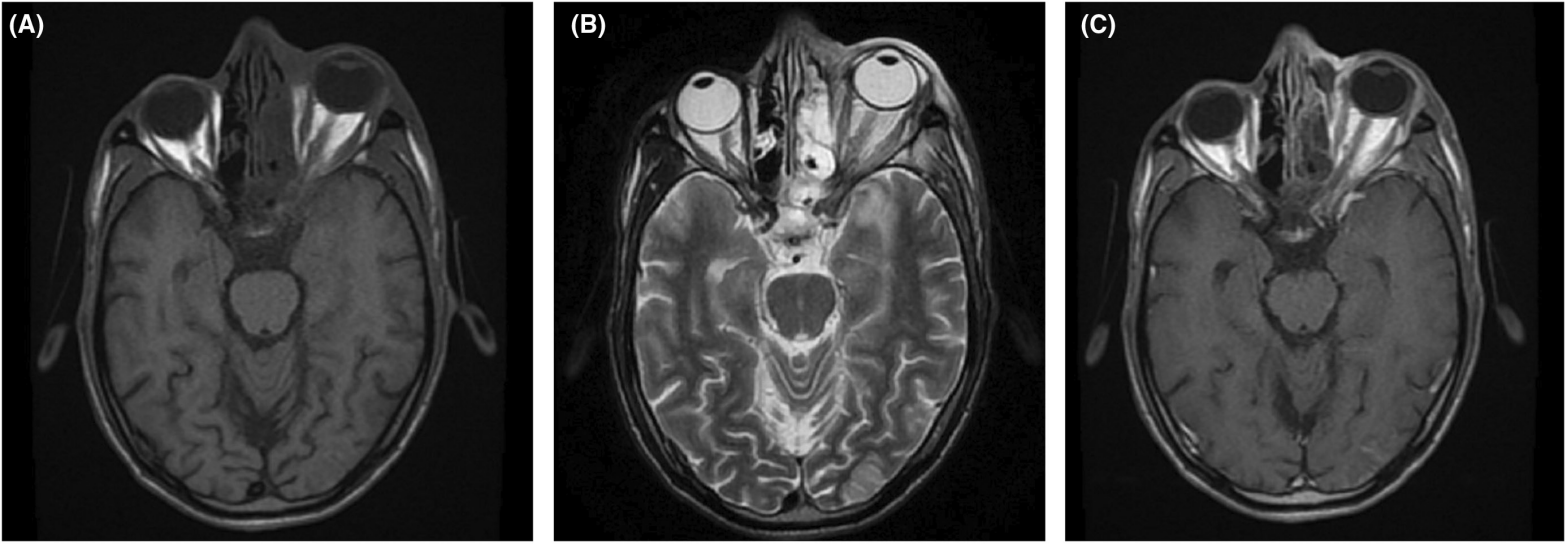
(A) Time to echo phase 1 (T1WI) shows left optic nerve thickening with no altered signals. (B) T2WI shows left optic nerve with altered T2 signals and shows mild thickening. (C) T1W Post‐contrast shows slight perineural enhancement with optic nerve thickening.

**FIGURE 2:**
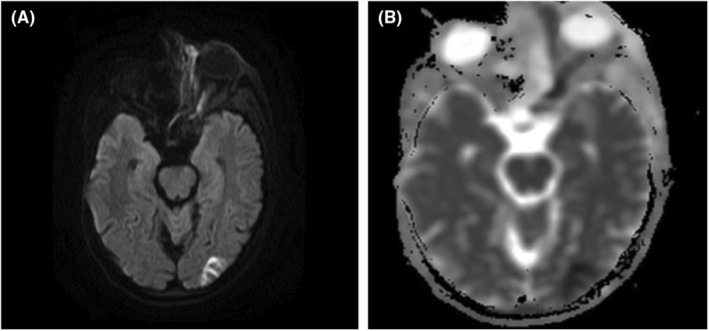
(A, B) Diffusion‐weighted imaging (DWI). (A) shows hyperintense signals within the left optic nerve, which correlates with hypointense signals on apparent diffusion coefficient (ADC) mapping (B), suggesting diffusion restriction consistent with optic nerve infarction. Further diffusion restriction is seen in the left occipital lobe suggesting an intracranial extension of the disease process.

**FIGURE 3:**
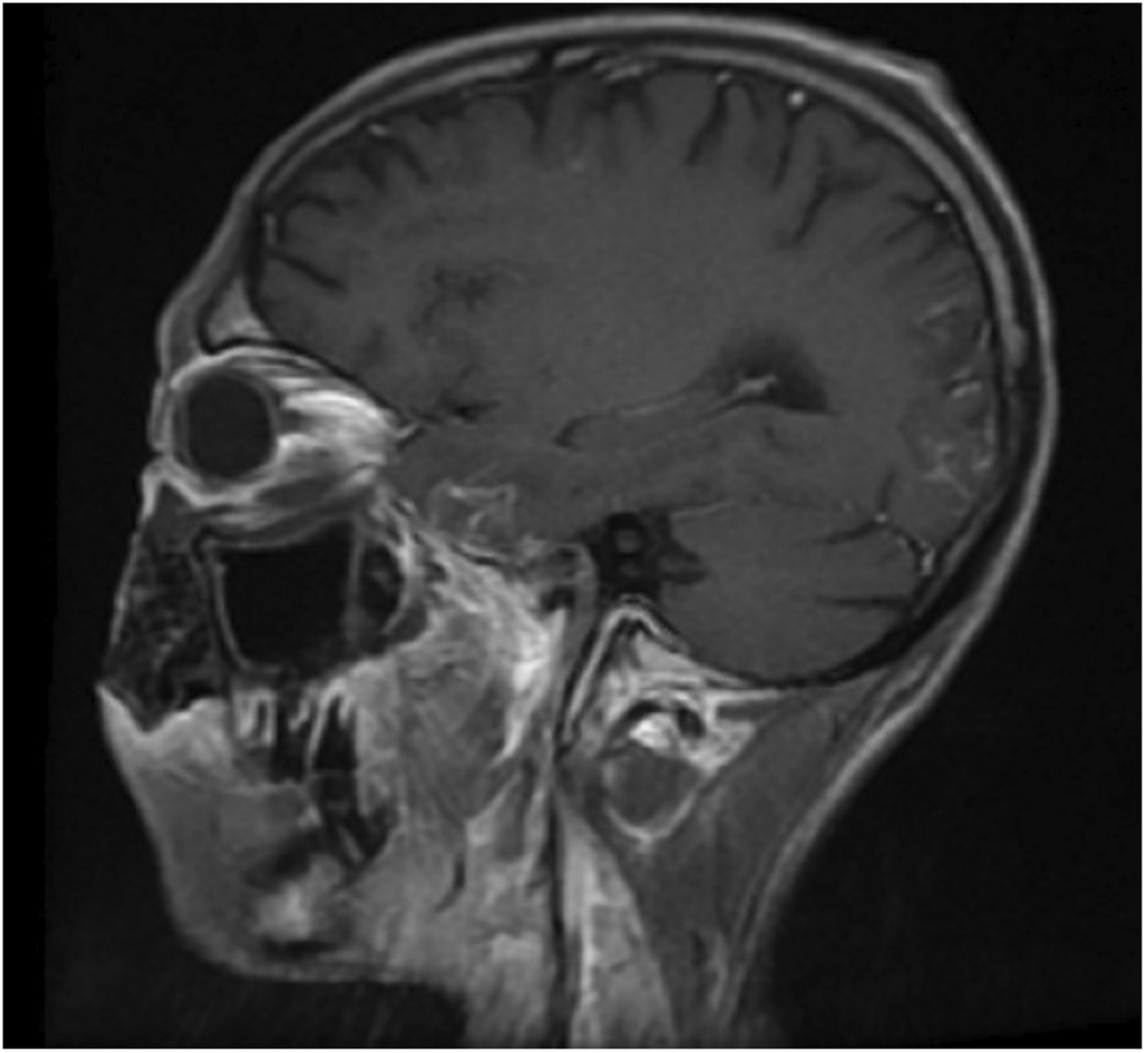
Time to echo phase 1 (T1WI) with contrast reveals interrupted leptomeningeal enhancement in left temporal and occipital lobes suggesting meningitis – sequelae of mucormycosis suggesting leptomeningeal involvement by the fungal infection.

A 35 years old male patient presented in the hospital with complaints of left‐sided facial swelling, blindness in the left eye, and left eye proptosis. He had a concomitant history of diabetic ketoacidosis (DKA). Magnetic resonance imaging (MRI) was advised. MRI findings were suggestive of rhino occulo cerebral mucormycosis. Mucor, a fungus, can lead to deadly infection of the paranasal sinuses, eventually involving the orbit and brain.^1^ There were only limited cases of diffusion‐weighted MRI demonstrating ischemic optic neuropathy when the literature was scoured.^2^ Figures 1, 2, 3 with description provide a comprehensive illustration.

## AUTHOR CONTRIBUTIONS

**Shehroze Tabassum:** Conceptualization; writing – original draft; writing – review and editing. **Aroma Naeem:** Writing – original draft. **Afshan Shakir:** Writing – original draft; writing – review and editing. **Faiza Afzal:** Writing – original draft. **Laya Ohadi:** Writing – original draft.

## CONFLICT OF INTEREST

None.

## CONSENT

Written informed consent was obtained from the patient for publication of this image report in accordance with journal's patient consent policy.

## Data Availability

The data and materials used in the current study are available from the corresponding author upon reasonable request.
